# Beyond discretization: why cognitive science should embrace continuity

**DOI:** 10.3389/fpsyg.2026.1777565

**Published:** 2026-04-24

**Authors:** Irene Di Pietro, Giada Viviani

**Affiliations:** 1 Padova Neuroscience Center, University of Padova, Padova, Italy; 2 Department of Developmental Psychology and Socialisation, University of Padova, Padova, Italy

**Keywords:** continuous modeling, continuous processes, discretization bias, semantic gradient, statistical learning

## 1 Introduction

Cognitive psychology has historically advanced by simplifying the mind into discrete contrasts—binary or multilevel (e.g., congruent/incongruent, low/medium/high)—to facilitate effect detection. While pragmatically useful, these discrete operationalizations are often mistaken for ontological truths, rarely reflecting the reality of the constructs they measure. Yet, following the “Law of Continuity” (Leibniz, 1996), continuity should be the theoretical default assumption: *Natura non facit saltus*. This principle is not limited to cognitive psychology but extends to the entire architecture of cognitive science, from the formalisms of artificial intelligence and the gradients of linguistic meaning to the continuous dynamics of social coordination and the fluid coupling of ecological agent-environment interactions.

The continuity of cognition is biologically undeniable but also computationally and representationally grounded. The brain encodes information through graded population codes, not binary switches; tuning functions map stimuli onto smooth probability distributions, enabling complex computations (Averbeck et al., 2006; Pouget et al., 2003). Consequently, neural representations are best described as vectors within continuous spaces rather than discrete states (Griffiths et al., 2010). Moreover, recent evidence indicates that even large-scale brain organization follows continuous gradients rather than sharply bounded units (Hayden et al., 2025).

This dictates a clear epistemological stance: dimensionality is the natural null hypothesis for psychological constructs (Meehl, 1992). Unless a categorical distinction (*taxon*) is explicitly proven, the default assumption must be that cognitive phenomena vary in degree.

Persisting with discrete frameworks for continuous phenomena sets a dual trap. Theoretically, it violates structural fidelity (Messick, 1995) misrepresenting continuous mechanisms as discrete steps, thus undermining validity (Borsboom et al., 2004). Methodologically, discretization reduces statistical power and masks the effect's functional form (e.g., Cohen, 1983; MacCallum et al., 2002; Young, 2016).

The field must therefore move beyond effect detection to precise estimation and modeling (Maxwell et al., 2008; Rodgers, 2010). Embracing continuity is essential for predictive, generalizable, and neurobiologically plausible theories. In this Opinion, we illustrate this shift through semantic representation and statistical learning, before briefly discussing continuity as a unifying principle across cognitive domains.

## 2 The geometry of meaning: from categorical distinctions to continuous spaces

Measurement must respect phenomenal structure (Messick, 1995). When theories describe continuous processes, their operationalizations should likewise be continuous. Interference resolution, linguistic interference particularly, offers a critical testbed for this necessary methodological and theoretical shift.

**Figure 1:**
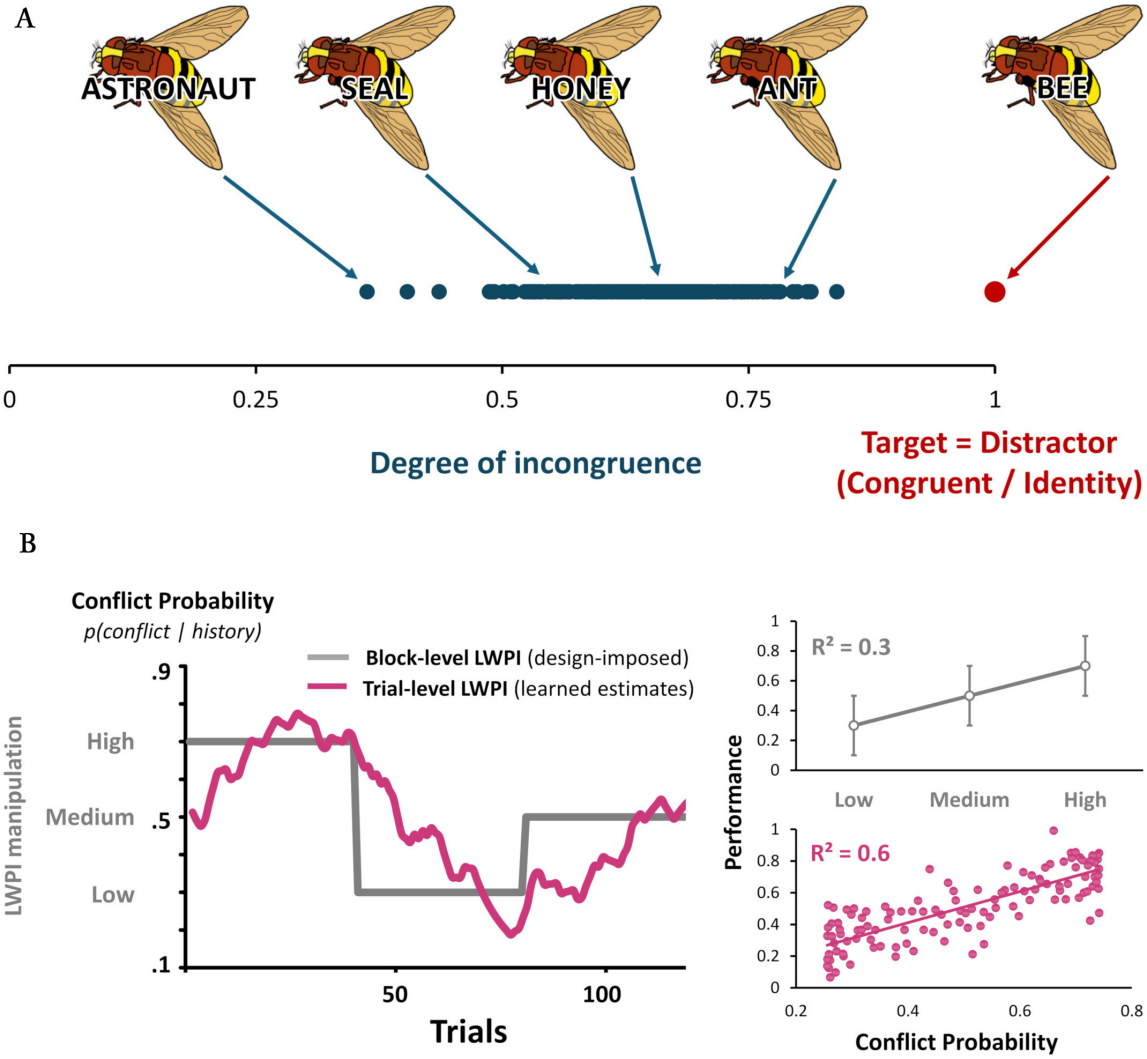
**(A)** Single representational configuration vs. continuous similarity gradient. Congruent picture–word pairs occupy a single representational configuration (identity; cosine similarity = 1). In contrast, incongruent pairs span a continuous similarity gradient, often empirically extending from near zero to less than 1. Yet binary congruency (congruent/incongruent), binary level of relatedness (related/unrelated) and ordinal levels (high/medium/low) equate subtle with substantial differences, imposing arbitrary cutoffs on a smooth continuum. Discrete distinctions remain useful in experimental design to sample semantic space, but the semantics operate over a continuous semantic landscape—best captured in analysis via continuous predictors (e.g., subjective ratings) or multidimensional similarity matrices (e.g., word embeddings), preserving the phenomenon's structure at the level where theoretical inference is drawn. Binary or multilevel contrasts collapse this manifold into discrete classes, masking the incongruency similarity space characterized by many possible degrees of mismatch. Example stimuli adapted from Duñabeitia et al. (2022)), licensed under CC BY 4.0, The Multilingual Picture Database. **(B)** Statistical learning in adaptive control. **(Left)** Temporal evolution of conflict probability (control expectations, *y*-axis) across trials (*x*-axis). Standard list-wide proportion incongruency (LWPI) designs manipulate conflict frequency via discrete blocks (gray step function), fixing the proportion of incongruent trials within each block. This manipulation defines discrete global conflict contexts (e.g., low, medium, high) that are constant within blocks. Block-level LWPI quantification assigns identical conflict probability to all trials within a block, assuming stable expectations. In contrast, trial-level LWPI (fuchsia line) represents a continuously updated estimate of conflict probability inferred from recent history. Following Viviani et al. (2024b)), this trial-level LWPI was obtained using the Hierarchical Gaussian Filter (Mathys et al., 2014) to extract dynamic trial-level conflict probability estimates from the sequence of conflict events (for a tutorial see https://osf.io/qmu7g). This estimate evolves gradually, fluctuates within blocks, and does not reset abruptly at block transitions, capturing learning-driven variations in control demands. **(Right)** Conceptual illustration of the impact on analysis. Performance (*y*-axis) improves with higher conflict probability. **(Top)** Block-level analyses treat LWPI as discrete (gray points), aggregating performance within block-defined conditions (Low, Medium, High) to then contrast blocks. By treating intra-block variation as noise, this approach explains only limited variance (illustrative *R*^2^ = 0.3). **(Bottom)** Trial-level[[Inline Image]] analyses model conflict probability continuously (fuchsia points; e.g., using linear mixed-effects models). This reveals that performance scales smoothly with dynamically learned conflict expectations. By capturing variance in control deployment driven by local fluctuations in probability, continuous modeling improves explanatory power (illustrative *R*^2^ = 0.6) and unveils the functional relationship between conflict expectation and adaptive control.

Since Stroop (1935)), interference has been studied primarily through binary contrasts (e.g., congruent/incongruent). Yet this dichotomy masks a fundamental property: while congruency is a single configuration, incongruency spans a continuous space of mismatches (Figure 1A). Consequently, the relationship between task-relevant and task-irrelevant dimensions is rarely binary. As Klein (1964)) observed, naming the ink color blue on the word “sky” creates more interference than on “house,” just as perceptual interference scales smoothly with hue similarity. These observations suggest that stimulus relations vary along a continuum of representational overlap; binary contrasts compress this structure, obscuring its underlying geometry.

This reductionism is particularly evident in semantic processing. Standard Picture–Word Interference experiments typically treat relatedness as a binary state (e.g., related dog–cat vs. unrelated dog–table; Korko et al., 2024), assuming concepts are either inside or outside a semantic category. Even designs introducing ordinal similarity levels (e.g., high/medium/low; Vigliocco et al., 2004; Vieth et al., 2014) impose arbitrary cutoffs that equate subtle and substantial differences (Figure 1A). Furthermore, this discretization fails to capture human cognition's ability to form flexible, goal-dependent groupings based on graded, *ad-hoc* similarity (e.g., “things that I can play with”).

Empirical evidence supports a continuous account. Picture-Word Interference effects scale linearly with fine-grained feature overlap (Aristei and Abdel Rahman, 2013), and mouse-tracking data reveals that motor trajectories are continuously shaped by semantic similarity (Gatti et al., 2024). These findings suggest competition is a matter of degree within a continuous landscape.

This aligns with recent theories viewing semantic memory as a dynamic system. Modern network, feature-based, and distributional models compute semantic similarity via vector proximity (Kumar, 2021). Furthermore, neural recordings reveal that neural populations encode information through continuous “embeddings” rather than discrete classes (Goldstein et al., 2024; Jamali et al., 2024). Importantly, acknowledging this gradient does not require abandoning categorical experimental designs, which remain useful for sampling the semantic space (see Discussion).

Ultimately, for neurobiologically grounded accounts, we should no longer ask *if* a distractor interferes, but *how* it deflects retrieval trajectories within a multidimensional space. As we argue next, this insight extends beyond semantics to statistical learning, where continuous dynamics—not discrete transitions—provides a more plausible account of brain predictions and adaptations.

## 3 The dynamics of adaptive control: from blocks to continuous expectations

Measurement must respect cognitive dynamics no less than representational geometry. Cognitive computations are themselves graded processes unfolding through continuous statistical learning. Predictive-processing frameworks formalize cognition as the incremental updating of probabilistic expectations (Friston, 2009). Here, the brain continuously integrates reliability-weighted evidence to minimize uncertainty and support inference (Knill and Pouget, 2004), dynamically adjusting learning rates to surprise and volatility (Nassar et al., 2010; Heilbron and Meyniel, 2019). Cognition thus operates through continuous expectation learning rather than shifts between discrete contextual states. Yet cognitive psychology often creates an ontological mismatch by freezing these dynamics into static block-level measurements.

Adaptive control exemplifies this mismatch. To effectively achieve goals, control is continuously adjusted to contextual demands inferred via statistical learning. Experience-based regularities (e.g., conflict frequency) are translated into expectations, driving graded control adjustments (Egner, 2023). These emerge gradually as experience accumulates trial-by-trial, rather than in response to discrete contextual states. To investigate this, researchers typically manipulate conflict probability using blocked designs to shape expectations and control levels (Figure 1B), for example varying the List-Wide Proportion of Incongruent trials (LWPI) in Stroop tasks. While effective in inducing a global conflict context, quantifying conflict probability as block-wise LWPI levels relies on two fallacies: it unrealistically assumes that the block's statistical structure is immediately available to participants, and that conflict probability—and thus control demands—is homogeneous across trials within a block. This ignores the learning process itself, implying that participants uniformly adjust to a context they are uninformed about and have not yet fully experienced, which is untenable. Although block-level measures yield robust effects (Bugg, 2017), treating conflict probability as a constant raises fundamental validity issues, failing to precisely model how conflict probability is inferred via statistical learning and averaging away the very learning mechanism that generates the behavior.

Instead, within LWPI blocks, conflict expectations update trial-by-trial as evidence from recent experience accumulates. Control adaptations track the predictive value of recent events (i.e., trial sequences)—a dynamically evolving expectation—rather than the experimenter-assigned block context (Jiang et al., 2014). Consequently, the “true” driver of control is not a static block-level condition, but a latent, trial-by-trial estimate of conflict probability that must be quantified at the level where learning occurs: the trial (Figure 1B).

This mismatch can be resolved by explicitly modeling the internal estimation process. For instance, Viviani et al. (2024b)) have employed Bayesian learning frameworks (i.e., Hierarchical Gaussian Filter; Mathys et al., 2014) to model LWPI as a continuously updated conflict probability estimate inferred from trial history (see also Sali et al., 2024; Figure 1B). Crucially, LWPI trial-level estimates explained behavior better than conventional block-level measures, validating continuous learning-based modeling of adaptive control. By dissociating experimental design from the internal variables governing behavior, this approach resolves the mismatch between discrete measurement and the graded, learning-based nature of adaptive control.

## 4 Continuity as a unifying cognitive principle

The two cases discussed so far illustrate how treating inherently continuous cognitive processes as discrete distorts theory and measurement. But they are not isolated exceptions. Rather, continuity is a unifying principle that extends across domains and even timescales.

At the intra-trial scale, binary responses (e.g., left/right) in perceptual decision-making reflect only the final readout of continuous internal dynamics. Evidence-accumulation models (Heekeren et al., 2008; Palmer et al., 2005; Voss et al., 2013), neural recordings from parietal and premotor cortices (Shadlen and Newsome, 2001; Cisek, 2007), and mouse-tracking studies (Spivey, 2007) show that decisions evolve through the gradual build-up of sensory evidence, cascading seamlessly into action and revealing real-time representational competition.

Expanding to the trial-by-trial scale, reward-guided behavior extends statistical learning to value-based domain. Humans continuously update expectations about reward probability and volatility (e.g., Behrens et al., 2007). Consistently, dopaminergic neurons do not signal reward presence/absence but encode graded prediction errors proportional to expectation violations (Schultz, 1997). Thus, adaptation via continuous updating generalizes beyond control to reinforcement learning.

Finally, continuity in learning extends to the lifespan scale, reflecting the cumulative integration of statistical regularities in vision. Such long-term priors tune not only basic perceptual mechanisms (e.g., Gestalt perceptual principles, color/shape constancy; e.g., Wagemans et al., 2012), but also high-level visual processes. Object–scene co-occurrences (e.g., a toaster in a kitchen) are naturally represented as graded likelihoods rather than binary congruent/incongruent contrasts (cf. Bar, 2004). Treating contextual consistency as a continuous probability could reveal that congruency effects are simply snapshots of an underlying graded predictive system. This represents a clear case in which probabilistic theorizing has not yet been matched by corresponding continuous operationalization (but see Di Pietro et al., 2025).

These examples confirm continuity as a core, unifying cognitive principle, which nonetheless has only been partially translated into continuous measurement practices. And this principle scales up from individual learning to collective dynamics. Recent frameworks in social predictive processing and variational neuroethology (Constant et al., 2019; Veissière et al., 2020) conceptualize social and cultural interaction as the continuous synchronization of probabilistic expectations between agents. By treating cultural patterns as graded distributions of shared priors rather than discrete categories, cognitive science can better model how meaning emerges and stabilizes across different scales of social organization.

## 5 Discussion

The central claim of this Opinion is straightforward: scientific methods must reflect the continuity of cognitive phenomena, which are graded, probabilistic, and dynamically evolving. Yet they are often operationalized, measured, and analyzed as discrete states. This mismatch is not merely technical; it compromises validity—structural fidelity—and obscures the very mechanisms our theories seek to explain (Borsboom et al., 2004).

While our discussion has focused on individual-level mechanisms, reflecting our areas of expertise, we argue that this ontological stance is equally vital for the broader multidisciplinary ambitions of cognitive science. Whether analyzing the dynamics of social interaction or language, or the performance of neural networks, a continuous framework prevents the reification of arbitrary boundaries and fosters a more integrated understanding of the mind.

The two case studies discussed here provide two illustrative slices within cognitive psychology to ground our claim that continuity is the default ontological property of cognitive processes, unless strong evidence justifies discreteness. When theoretically continuous constructs are modeled accordingly, explanatory power increases and previously obscured latent structures become visible.

This continuity principle has direct implications for measurement. The core issue arises when measures used to index cognitive phenomena do not respect their latent structure. Discrete experimental manipulations—such as blocked designs or binary contrasts—can be legitimate and useful probes. Problems emerge when these design choices are reified as properties of the cognitive system itself. In semantic paradigms, “related” and “unrelated” distractors conveniently sample a graded similarity space, but cognition operates over that entire space. In adaptive control, block-wise manipulations of conflict frequency effectively provide statistical regularities, but expectations—and the control adaptations they drive—evolve through trial-by-trial learning. Measures that inherit the discreteness of the design as a theoretical commitment therefore risk mischaracterizing the phenomenon under study. Instead, measures should explicitly capture the underlying continuous structure—using trial-level estimates or continuous predictors.

Beyond alignment with latent structure, continuous modeling offers substantial inferential advantages. Discretization is well-known to reduce statistical power and measurement reliability and obscure the functional form of the relationship (MacCallum et al., 2002; Young, 2016; Cohen, 1983). Sampling only two points along a continuum (e.g., Low vs. High) leaves many competing theories observationally indistinguishable. Continuous approaches, instead, allow researchers to map the full gradient—using tools like mixed-effects or Bayesian hierarchical models (Viviani et al., 2024a)—enabling theories to specify exactly how behavior should change with the underlying dimension. In this sense, continuous modeling increases falsifiability: it tightens the link between theoretical structure and observable data.

Accordingly, continuity should be treated as the natural null hypothesis for psychological constructs. Conceptualizing constructs as discrete or continuous carries profound epistemological implications: when scientific methods fail to model this continuity, they risk mistaking measurement artifacts for cognitive structure. Any decision to discretize must therefore be justified (Meehl, 1992) by both theoretical goals and statistical implications. Discrete distinctions should be treated as hypotheses to be tested, not assumptions: if discontinuities exist, they should emerge empirically as sharp transitions or attractor boundaries within an otherwise continuous space. Treating variables as discrete from the outset precludes evaluating whether a discrete representation is warranted.

While our discussion focused on individual representational and control mechanisms, the principle of continuity has profound implications for the broader “neomechanistic” turn in cognitive science. From this perspective, cognitive mechanisms—whether confined to neural circuits or distributed across agents and environments (Hutchins, 1995)—are best understood as dynamical systems operating over continuous state-spaces. Adopting continuity as an ontological default provides the formal language necessary to bridge neural and psychological data with the insights of cognitive anthropology and situated cognition, fostering a truly integrated and non-reductive science of the mind.

Identifying robust effects has been a major achievement of cognitive science, yet relying on existence proofs is no longer enough. We must now model how these effects emerge and evolve. Aligning measurement with the continuous nature of cognitive processes is not a mere refinement, it is a prerequisite to enhance validity, statistical power and reliability, and falsifiability. Only by treating continuity as the default can we transform static descriptions into predictive, neurobiologically grounded theories. Continuity is not a reductionist move; on the contrary, it is the only framework capable of capturing the seamless transition between neural activity, individual behavior, and the distributed dynamics of social and cultural systems. The world is rarely black and white—and neither is the mind.
